# Measurements of enteral feeding intolerance in critically ill children: a scoping review

**DOI:** 10.3389/fped.2024.1441171

**Published:** 2024-10-10

**Authors:** Yan Li, Cong-Hui Fu, Min-Jie Ju, Ji Liu, Xiao-Ya Yang, Ting-Ting Xu

**Affiliations:** ^1^ Department of Critical Care Medicine, Shanghai Children's Hospital, Shanghai, China; ^2^ Department of Critical Care Medicine, Shanghai Children's Hospital, School of Medicine, Shanghai Jiao Tong University, Shanghai, China; ^3^ Department of Nursing, Shanghai Children's Hospital, Shanghai, China; ^4^ Department of Nursing, Shanghai Children's Hospital, School of Medicine, Shanghai Jiao Tong University, Shanghai, China

**Keywords:** critical care nursing, child, enteral nutrition, feeding intolerance, symptom assessment, review

## Abstract

**Objective:**

To examine the measurements on enteral feeding intolerance (EFI) in critically ill children.

**Methods:**

The Joanna Briggs Institute methods for conducting a scoping review were followed. Articles published since 2004 which assessed EFI in critically ill children were identified. A full search strategy was executed in seven English databases (MEDLINE, EMBASE, PubMed, Web of Science, Cochrane Central Register of Controlled Trials, JBI EBP, CINAHL) and four Chinese databases (CNKI, VIP, Wanfang, Sinomed). Two reviewers screened records according to our inclusion and exclusion criteria, and conducted a full-text review of selected articles. The reference lists of all studied selected were screened for additional sources. Relevant data was extracted using a researcher-developed tool.

**Results:**

Of the 627 articles identified, 32 were included in this scoping review. Most articles focused on the measurement of high gastric residual volume (*n* = 22), followed by diarrhea (*n* = 20), and vomiting (*n* = 9). Most of the studies were of observational-analytic design (13/32) and experimental design (8/32).

**Conclusion:**

This scoping review addressed the complexity and diversity of EFI measurements. Given the importance of adequacy of enteral nutrient intake, we highlighted the necessary to develop individual measurements of EFI, taking the age of children and disease condition into consideration. Further studies can also investigate accurate and objective physiological measurements of EFI to advance EN and improve outcomes in critically ill children.

## 1 Introduction

Malnutrition has been recognized as a major health problem in pediatric intensive care unit (PICU), which affects 37.19% of critically ill children (1). On PICU admission, 18% to 47% (2, 3) of critically ill children are already malnourished. During the hospitalization in PICU, critically ill children often go through a catabolic stress state and altered inflammatory response due to trauma of infection (4, 5), and 74% of them may experience iatrogenic underfeeding encouraged by prolonged fasting and frequent feeding pauses (6). Meanwhile, taking the lower percentage of muscle mass and fat, higher resting energy expenditure (REE) and great nutritional requirements for growth and development (7) into consideration, nutritional deterioration is frequent and often intense. In critically ill children, malnutrition is associated with deterioration of muscle strength, multiorgan dysfunction, increased risk of infection, greater length of mechanical ventilation (MV) and PICU stay, and increased mortality (7–10). The relationship between nutrition support therapy and the improvement in clinically relevant outcomes in pediatric critical care has currently demonstrated by advances in published scientific literature (11). Optimal nutritional support therapy can avoid under or overfeeding, showing advantages in attenuating the morbidity rate, decreasing the length of PICU stay, and improving patient outcomes (12).

Among several ways to provide nutritional support therapy, enteral nutrition (EN) is recommended as the preferred nutrition mode in critically ill children by Society of Critical Care Medicine (SCCM) and American Society for Parenteral and Enteral Nutrition (ASPEN) (13). EN is proved to have many advantages, such as inducing gastrointestinal mucosa trophism to avoid bacterial translocation, costing less hospitalization expenses and decreasing the risk of infection than parenteral nutrition (PN) (14). Additionally, achievement of up to two thirds of the nutrient goal (15) and withholding PN (16) in the first week of critical illness is associated with improved outcomes. Thus, European Society of Pediatric and Neonatal Intensive Care (ESPNIC) recommends using EN protocols to initiate nutrition delivery in 24 h and improve nutritional intake to reduce the risk of malnutrition and promote recovery (15). However, more than half of children cannot receive adequate energy or protein intake through EN at their first week of admission (17, 18). Among the obstacles inhibiting the delivery of optimal EN, enteral feeding intolerance (EFI) is one of the main reasons (19), and the detecting of EFI should be included in the EN protocol (13).

EFI can result in feed interruptions, leading to insufficient energy delivery and delayed achievement of EN (20), and is also associated with skin integrity, pulmonary infections, and sepsis (21). However, the concept of EFI in critically ill children remains nebulous and inconsistent. It is usually characterized by clinical symptoms, with a 20% incidence in PICU (22). The most common clinical symptoms include increased gastric residual volume (GRV) and gastrointestinal (GI) discomfort signs, such as epigastric discomfort, vomiting, diarrhea, reflux, abdominal distension and pain/discomfort (23). However, the measurements of EFI are different across different PICUs, and sometimes even across the same PICU. The time point, frequency and threshold of GI symptom monitoring vary among different studies (23). The difference in measurements can affect the detection of EFI, and it is likely that the subjective measurements, which lack an evidence base, are conservative and define a lower threshold for withholding of EN than necessary (24). This may cause unnecessary nutritional interruption, and contribute to increased risk of inadequate nutrient intake.

Therefore, a comprehensive review summarizing the measurements of EFI in critically ill children is essential from a clinical and scientific perspective. Given the heterogeneity of EFI definitions in studies, we cannot draw any firm conclusions on measurements, prevalence or outcomes through a systematic review (22). Under this circumstance, a scoping review can be suitable to investigate research involving the measurements of EFI. Thus, the objective of the present scoping review was to systematically map out the body of existing literatures on measurements of EFI in critically ill children to identify knowledge gaps and opportunities for further research.

## 2 Materials and methods

### 2.1 Protocol and registration information sources

This review was conducted in accordance with the Joanna Briggs Institute methodology for scoping reviews, and has been registered with Open Science Framework (osf.io/5hbe7). The review was completed in a systematic, rigorous, and transparent way to minimize bias. We performed this scoping review of literature to summarize the measurements of EFI in pediatric critically ill population.

### 2.2 Search strategies

A comprehensive search of the literature was conducted through 7 English electronic databases and 4 Chinese electronic databases: MEDLINE, EMBAS, PubMed, Web of Science, Cochrane Library, JBI EBP, CINAHL, CNKI Citation, VIP, Wanfang Medical Network, and Sinomed. As a first step, a limited research combing MeSH terms and keywords was conducted in the databases of PubMed and CNKI, we piloted the searching strategy to check the appropriateness of the keywords and databases. The initial search terms in PubMed included (child OR adolescent OR pediatrics) AND (critical care nursing OR critical illness) AND (enteral nutrition OR enteral feeding) AND (symptom assessment OR measurements). Suitable articles were examined, with keywords and index terms identified from titles and abstracts (e.g., preschool child, teenager, pediatric intensive care unit, artificial feeding, symptom evaluation, etc.) used to develop a full search strategy for PubMed and CNKI databases, which can be modified and adapted to suit a range of databases in English and Chinese. Thereafter, a second search using all the identified MeSH terms, keywords and index terms was done across all databases. We searched the PubMed database with the following strategy: (((([child OR children OR preschool child* OR adolescent* OR adolescence OR teenager* OR youth* OR teen* OR pediatric* OR paediatric*(Title/Abstract)] OR [child OR child, preschool OR adolescent OR pediatrics(MeSH Terms)]) AND ((critical care nursing OR intensive care units, pediatric OR intensive care units OR critical illness OR intensive care nursing OR critical care OR intensive care unit OR pediatric intensive care unit* OR Pediatric ICU OR PICU OR ICU OR critically ill OR critical illnesses[Title/Abstract]) OR (critical care nursing OR intensive care units OR intensive care units, pediatric OR critical illness[MeSH Terms]))) AND ((enteral nutrition OR nutritional support[MeSH Terms]) OR (enteral nutrition OR nutritional support OR enteral feeding OR tube feeding OR gastric feeding tube OR nutrition support OR artificial feeding [Title/Abstract]))) AND (enteral feeding intolerance OR complication OR feeding intolerance OR enteral nutrition intolerance[Title/Abstract])) AND ((symptom assessment OR nursing assessment OR symptom assessments OR symptom evaluation OR nursing assessments OR assess OR measurements[Title/Abstract]) OR (symptom assessment OR nursing assessment[MeSH Terms])). Our research was limited to articles published from 1 January 2004 until 31 December 2023, as the first review paper of feeding intolerance in children was published in 2004. We performed the searches on April 12, 2024. The full search, as executed, is available in Supplementary File 1. Additionally, the third step included screening of the reference lists of all studies selected for this scoping review to look for additional sources.

### 2.3 Inclusion and exclusion criteria

The inclusion criteria for our study were: (1) the population consists of pediatric patients (28 days to 18 years old (13), with critical illness; (2) the article includes detailed methods for measuring EFI, including delayed gastric emptying (GE) and GI symptoms (nausea, vomiting, diarrhea, abdominal discomfort, etc.); (3) the types of studies include systematic reviews, experimental studies, observational studies, and qualitative study designs; (4) literature published in Chinese or English between 1 January 2004 and 31 December 2023. The exclusion criteria were (1) full-text vision unavailable; (2) opinion pieces and conference abstracts (e.g., editorials). Final results of the evidence search and selection progress were presented in a Preferred Reporting Items for Systematic Reviews and Meta-Analyses (PRISMA) flow diagram.

### 2.4 Data screening and extraction

All reference identified were imported into the reference manager software, EndNote X9. The references from different databases were combined and any duplicate records were removed. A two-steps screening process to select the study was adopted. Firstly, the title and abstract of study were screened, followed by a full-text review. Two reviewers screened the articles against the eligibility criteria independently. All the disagreements were discussed, and a third reviewer was consulted if no consensus can be reached. Relevant data were extracted from all included studies by two reviewers independently. A structured data recording form developed by the group was used and the information was recorded on Microsoft Excel. The following data were extracted: study design, study objective, study population, and measurements of EFI. Any disagreement between reviewers was resolved through discussion and a third review author acted as an arbiter when disagreements could not be resolved.

### 2.5 Data analysis and synthesis

In keeping with the primary intention of this scoping review to investigate and describe the available literature, analysis of the data gathered were descriptive in nature. The categorical elements of the data were counted and tabulated into groupings, such as the study design and country of origin. Qualitative data was organized into categories, and simple descriptive statistics, such as frequency counts and basic coding was used to collate quantitative data.

We divided the indicators of EFI into 2 categories: delayed gastric emptying (GE) and GI symptoms. Given the different descriptions in different studies, we combined terms with the same connotation. For example, vomiting also stood for emesis, and high GRV also stood for gastric retention. With each section, a narrative summary was completed to highlight the trends, gaps, and areas that warrant further study.

## 3 Results

### 3.1 Search results

**Figure 1:**
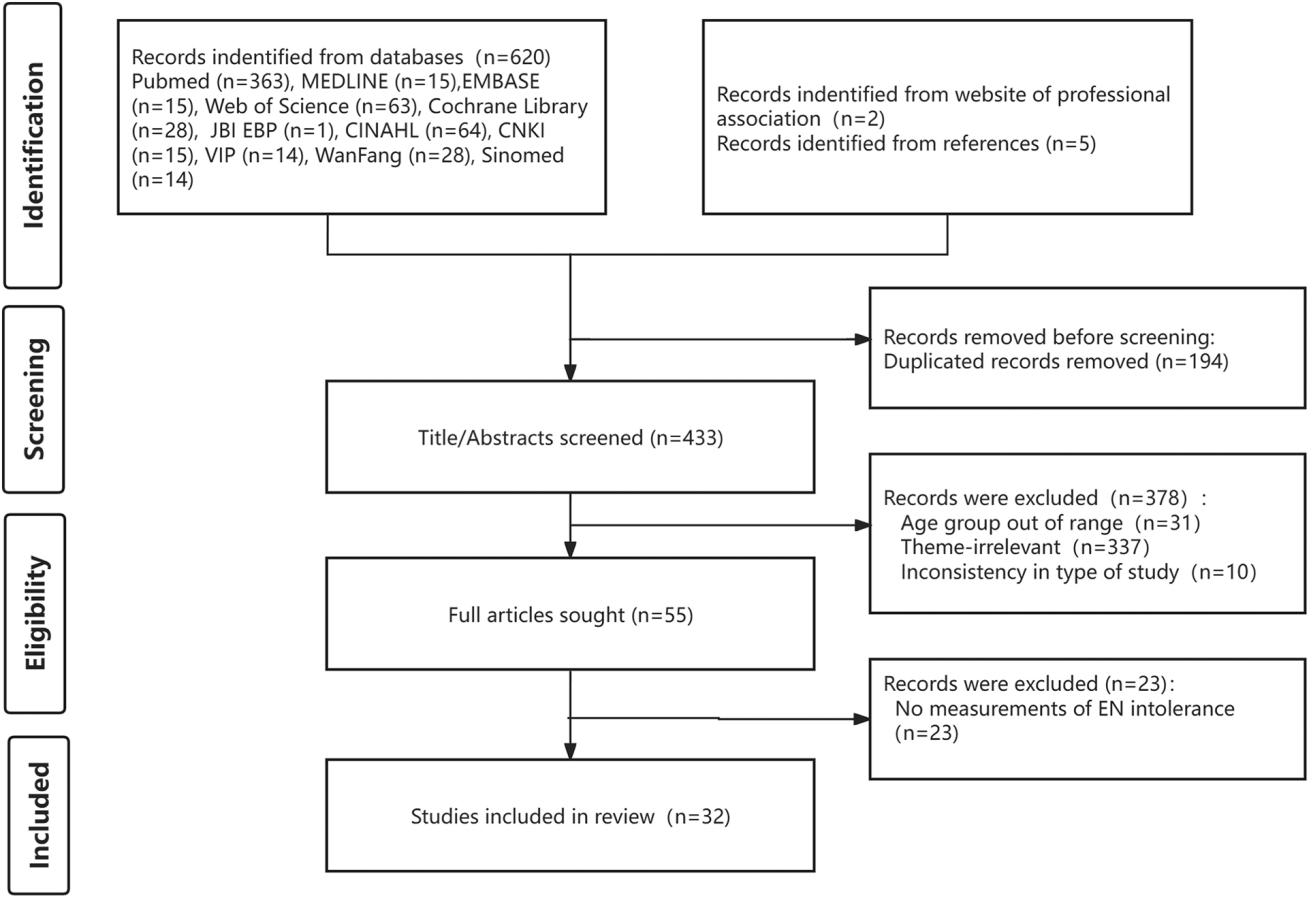
Flow diagram of the study selection.

In total, 627 results were obtained from the preliminary search. After removing duplicates, 433 articles were left for the screening of title and abstract. As a result, 55 articles met the inclusion criteria were retrieved to screen the full papers. 32 articles were included in the final data analysis. Figure 1 shows the screening process and results as a flowchart using the PRISMA template.

### 3.2 Study characteristics

Among the 32 included articles, 13 articles took the observational-analytic design, followed by experimental design (8/32) and quasi-experimental design (6/32). Seven studies were conducted in children with MV. Over half of the studies (17/32) were published in the last five years. The countries with the most publications are the United States (9/32), followed by China (8/32) and Spain (6/32).

### 3.3 Measurements of EFI

**Figure 2:**
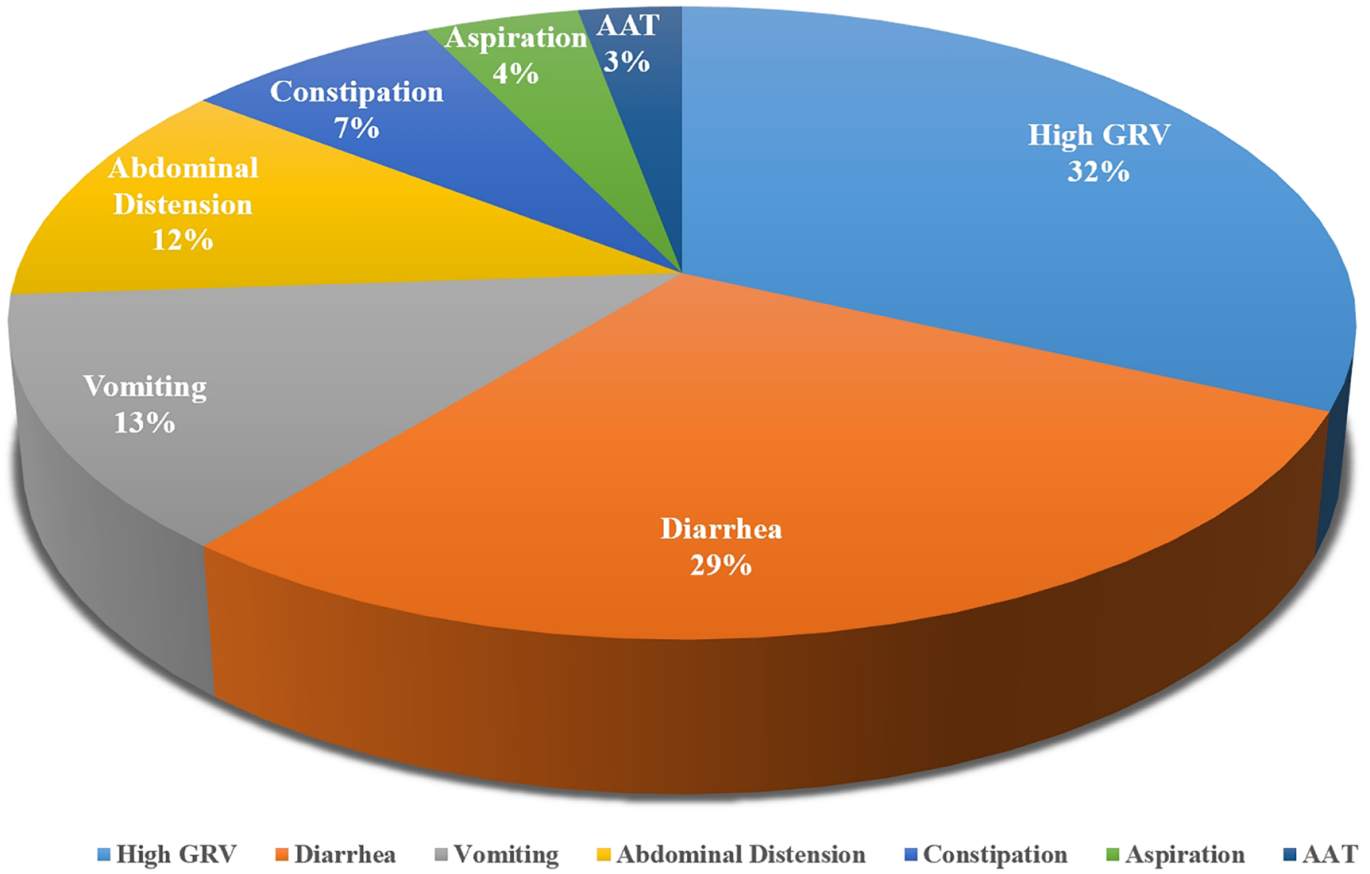
Indicators of EFI in critically ill children.

Different measurements were used in different studies. According to the characteristics of the indicators, we divided them into delayed GE and GI symptoms. The most common indicators included were high GRV, diarrhea, and vomiting. The summaries of the measurements were shown in Table 1. In the 32 included studies, a total of 7 indicators of feeding intolerance were identified, with measurements mentioned 69 times. The frequency of mentions for each indicator is shown in Figure 2.

**Table 1:** Indicators and corresponding measures of EFI in critically ill children.

Author	Region (year)	Journal	Objectives	Design	Population	Indicators of EFI and measures
Eveleens et al. (22)	Netherlands (2020)	Clinical Nutrition	To investigate the definitions, prevalence, predictors and outcomes of FI in critically ill children	Systematic review	Critically ill children	(1) High GRV:≥50% of the EN delivered in the last 4 h (2) Diarrhea: ≥4 times loose or liquid stool, with negative fluid balance in 24 h period (3) Vomiting: ≥2 times with gastric content in 24 h period
Ying et al. (25)	China (2023)	European Journal of Pediatrics	To understand the characteristics of children with FI and identify the factors predicting FI in critically ill children	Retrospective Cohort	Critically ill children	(1) High GRV: ≥50% of the EN delivered in the last 4 h (2) Diarrhea: ≥4 times loose or liquid stool, with negative fluid balance in 24 h period (3) Vomiting: ≥2 times with gastric content in 24 h period
Pérez et al. (26)	Spain (2022)	Journal of Pediatric Gastroenterology and Nutrition	To assess the safety of enteral nutrition in children on extracorporeal membrane oxygenation	Retrospective cohort	Pediatric patients on extracorporeal membrane oxygenation	(1) High GRV: ≥50% of the EN delivered in the last 4 h (2) Diarrhea: >8 liquid stools in infants <3 months of age, >4 liquid stools in 3–12-month-old children, >2 liquid stools in children >12 months (3) Abdominal Distension: increased abdominal circumference on the sagittal plane (4) Constipation: ≥ 3 days without bowel movement after the start of EN
López-Herce et al. (27)	Spain (2008)	European Journal Of Clinical Nutrition	To study the risk factors for gastrointestinal complications related to enteral nutrition in critically ill children.	Prospective cohort	Critically ill children	High GRV:≥50% of the EN delivered in the last 4 h
López-Herce et al. (28)	Spain (2008)	Nutrition Journal	To analyze the characteristics of enteral nutrition and its tolerance in the critically ill child with shock and to compare this with non-shocked patients	Prospective cohort	Critically ill children	High GRV:≥50% of the EN delivered in the last 4 h
van Waardenburg et al. (29)	Netherlands (2009)	Clinical Nutrition	Explore the effect of protein and energy-enriched infant formulas in achieving nutritional targets	RCT	Critically ill children	(1) High GRV: ≥50% of the EN delivered in the last 4 h (2) Diarrhea: ≥4 times loose or liquid stool, with negative fluid balance in 24 h period
Sánchez et al. (30)	Spain (2007)	Nutrition	To compared the tolerance of early and late transpyloric enteral nutrition in critically ill children	Prospective cohort	Critically ill children	(1) High GRV: ≥50% of the EN delivered in the last 4 h (2) Diarrhea: ≥5 loose stools per day
Yuqing et al. (31)	China (2021)	Journal of Nursing Science	To compare the sensitivity and specificity of different process assessment indicators or combinations of indicators in the diagnosis of feeding intolerance in critically ill children	Retrospective Cohort	Critically ill children	(1) High GRV: ≥50% of the EN delivered in the last 4 h (2) Vomiting: ≥2 times with gastric content in 24 h period (3) Abdominal Distension: increase in abdominal circumference ≥10% on 2 consecutive occasions within 24 h
Chiusolo et al. (32)	Italy (2020)	Pediatric gastroenterology, hepatology & nutrition	To assess the effectiveness and safety of amoxicillin/clavulanate (A/C) to treat EN intolerance	Quasi-experimental Study	Critically ill children	(1) High GRV: in continuous EN delivery, GRV ≥50% of the volume/h at least 3 consecutive evaluations, in intermittent EN delivery, GRV ≥50% of the bolus volume at least 3 consecutive evaluations (2) Diarrhea: ≥3 liquid stools/day in patients with previous normal stool and/or increase of ≥50% of the number of liquid stools
Bartkowska-Śniatkowska et al. (33)	Poland (2015)	Anaesthesiology Intensive Therapy	In the present study, methods for nutritional treatments in critically ill children are presented, depending on the clinical situation	Consensus	Critically ill children	(1) High GRV: in intermittent EN delivery, GRV should be measured before each bolus or every 4 h, GRV >5 ml/kg or over 50% of volume of the portion administered or 200 ml (in children with body weight >40 kg); in continuous EN delivery, GRV ≥200% of hour volume (2) Diarrhea: ≥4 loose stools/day (3) Constipation: >48 h without feces after the start of EN
Liauchonak et al. (34)	USA (2023)	Nutrition in Clinical Practice	To examine whether revising the EN intolerance definition of an algorithm would decrease EN interruptions and improve nutrient delivery in critically ill children.	Quasi-experimental Study	Critically ill children	High GRV: for patients >50 kg, GRV >250 ml, for patients <50 kg, GRV >3 ml/kg
Martinez et al. (35)	USA(2017)	Journal of Parenteral and Enteral Nutrition	To examine the correlation between (a) bedside EN intolerance assessments, including gastric residual volume (GRV); (b) delayed GE; and (c) delayed EN advancement	Prospective Cohort	Critically ill children	(1) High GRV: GRV >3 ml/kg or >150 Ml (2) Acetaminophen Absorption Test: A baseline acetaminophen level was obtained, and it was re-measured at 60 ± 5mins from acetaminophen administration (3) Diarrhea: ≥3 episodes of loose or liquid stool in a 24-h period (4) Vomiting: ≥2 times with gastric content in 24 h period (5) Abdominal Distension: 2 or more increases in abdominal girth in a 24-h period
Veldscholte et al. (36)	Canada (2023)	Journal of Pediatric Gastroenterology and Nutrition	To investigated the course of several gastrointestinal biomarkers and their association with EN advancement longitudinally during pediatric intensive care unit admission.	RCT	Critically ill children	High GRV: ≥50% of delivered EN over 24 h
Solana et al. (37)	Spain (2023)	Nutrients	To describe the characteristics of Enteral nutrition interruption in the pediatric intensive care unit	Observation	Critically ill children	High GRV: ≥50% of delivered EN over 24 h
Xianrong et al. (38)	China (2020)	The heart surgery forum	To explore the effects of breast milk feeding and formula milk feeding on infants after cardiac surgery in the cardiac intensive care unit	Retrospective cohort	Infants after cardiac surgery in ICU	(1) High GRV: in continuous EN delivery, GRV more than 50% of the previous total feeding amount (2) Diarrhea: defecation multiple times a day, mostly in the morning or after feeding, and mushy and watery stool, with a pungent odor
Wong et al. (25)	Singapore (2016)	Asia Pacific journal of clinical nutrition	To survey the nutrition practices and perspectives of paediatric intensivists and dieticians in Asia-Pacific and the Middle East	Observation	Critically ill children	High GRV: GRV >5 ml/kg or in >50% of the last feed volume
Yanqin et al. (39)	China (2018)	Nutrition	To evaluate nutrition effects and tolerance of a PE-formula compared with the standard formula (S-formula) in infants in the first 5 days after congenital heart surgery	RCT	Infants following congenital heart surgery	(1)
Shuangyu et al. (40)	China (2023)	China journal of Primary Medicine and Pharmacy	To explore the intervention effect of enteral nutrition tolerance management program in children with severe sepsis	RCT	Critically ill children	(1) High GRV: in continuous EN delivery, GRV >40% of the previous amount of milk pumped (2) Diarrhea: ≥1 time every 12 h (3) Vomiting: ≥1 time every 12 h (4) Abdominal Distension: intra-abdominal pressure over 10 mmHg (1 mmHg = 0.133 kPa) (5) Aspiration: Suction of stomach contents from the respiratory tract
Xianmin et al. (41)	China (2018)	China Medical Herald	To investigate the effect of enteral nutrition tolerance management program on early EN tolerance in children with severe sepsis	RCT	Critically ill children	(1) High GRV: in continuous EN delivery, GR V>40% of the previous amount of milk pumped (2) Diarrhea: ≥ 1 time every 12 h (3) Vomiting: ≥1 time every 12 h (4) Abdominal Distension: intra-abdominal pressure over 10 mmHg (1 mmHg = 0.133 kPa) (5) Aspiration: Suction of stomach contents from the respiratory tract
Huimin et al. (42)	China (2022)	China Medical University	By compiling and applying the questionnaire of knowledge, attitude and practice (KAP) of nurses in Pediatric Intensive Care unit (PICU) on enteral nutrition feeding intolerance, to explore the current level and influencing factors	Quasi-experimental Study	Critically ill children	(1) High GRV: GRV >1/3 of the previous feeding volume (2) Diarrhea: ≥6 loose stools per 24 h (3) Vomiting: ≥3 times/day (4) Abdominal Distension: abdominal circumference increase >1.5 cm in 24 h, with intestinal type
Valla et al. (43)	France (2022)	Frontiers in Pediatrics	To explore the effect of point-of-care ultrasound among pediatric intensivists.	Prospective Cohort	Critically ill children	High GRV: Scan gastric antrum larger and shorter diameters in a supine position and a right lateral decubitus position, to calculate the cross-sectional area of the antrum and extrapolating the gastric content volume based on the formula proposed by Spencer. The gastric content was described as “empty” or ‘full with liquid’ or ‘full with both solid and liquid’
Kaile et al. (44)	China (2023)	Chinese Pediatric Emergency Medicine	To review the progress on feeding intolerance and the relationship between gastric residual volume and feeding intolerance	Review	Critically ill children	(1) High GRV: In children ≤12 months, the formula proposed by Kim is more appropriate. The gastric volume can be delivered into 3 levels according to the presence of fluid in the gastric sinus in supine position or the right lateral decubitus position (2) Acetaminophen Absorption Test: A baseline acetaminophen level was obtained, and it was re-measured at 60 ± 5mins from acetaminophen administration to calculate the area under the curve at 60 min (AUC_60_). AUC_60_ <600mcg·min/ml is identified as delayed GE
Hamilton et al.(45)	USA (2014)	Pediatric Critical Care Medicine	To evaluate the impact of implementing an enteral nutrition algorithm on achieving optimal enteral nutrition delivery in the PICU	Quasi-experimental Study	Critically ill children	(1) Diarrhea: ≥3 episodes of loose or liquid stool in a 24-h period (2) Vomiting: ≥2 times with gastric content in 24 h period (3) Abdominal Distension: 2 or more increases in abdominal girth in a 24-h period
Kumar et al. (46)	India (2023)	Indian journal of pediatrics	To compare the time taken to reach the target calories and proteins by protocol based “continuous tube feeding” and “intermittent tube feeding” in critically ill children.	RCT	Critically ill children	(1) Diarrhea: ≥ 3 episodes of loose or liquid stool in a 24-h period (2) Abdominal Distension: >10% increase from baseline girth
Weckwerth et al. (47)	USA(2004)	Nutrition in Clinical Practice	To describes commonly used monitors for tolerance to enteral nutrition for infants and children and discusses pertinent data relevant to practice	Review	Critically ill children	Diarrhea: ≥3 episodes of loose or liquid stool in a 24-h period
Meert et al. (48)	USA (2004)	Chest	To determine the effect of feeding tube position (gastric vs small bowel) on adequacy of nutrient delivery and feeding complications, including microaspiration, in critically ill children	RCT	Critically ill children	(1) Diarrhea: ≥3 episodes of loose or liquid stool in a 24-h period (2) Aspiration: Aspiration was assessed by the detection of gastric pepsin in tracheal secretions. Tracheal secretions (0.1–0.5 ml) were collected from the endotracheal tube daily without the use of saline solution lavage
Panchal et al. (49)	USA (2016)	Journal of parenteral and enteral nutrition	To evaluate the safety of enteral feeding in children receiving vasoactive agents	Retrospective cohort	Critically ill children	Diarrhea: ≥3 loose watery stool in a day; for infants, it is defined as the passage of stool at least 1.5–2 times more frequent than the baseline level
Brown et al. (50)	USA (2012)	ICAN Infant, Child, & Adolescent Nutrition	To explore that a protocolized continuous gastric EN approach would decrease time to goal feeding rate and calories	Quasi-experimental Study	Critically ill children	Diarrhea: ≥6 loose stools per 24 h
Jacobs et al. (51)	USA (2013)	Pediatric Critical Care Medicine	To evaluate the impact of such an approach on the alteration of plasma phospholipid fatty acid concentrations.	RCT	Critically ill children	Diarrhea: >20 cc/kg/day of stool
Solana et al. (52)	Spain (2021)	Nutrition	To analyze the nutritional status, NS characteristics, macronutrient supply, and associations between NS and outcomes in critically ill children in Spain	Prospective cohort	Critically ill children	Constipation: ≥3 days without bowel movement after the start of EN
Marino et al. (53)	UK (2019)	Journal of human nutrition and dietetics	To characterise the use of a PEF amongst critically ill infants in two paediatric intensive care units	Retrospective Cohort	Critically ill children	Constipation: ≥4 days without stools
Brown et al. (54)	USA (2019)	Journal of parenteral and enteral nutrition	To compare the effectiveness and safety of C-GF vs B-GF in intubated pediatric patients	Quasi-experimental Study	Critically ill children	Constipation: >24 h without stools

#### 3.3.1 Delayed GE

Our review found that GE is measured by evaluating GRV and acetaminophen absorption test (AAT). 68.75% (22/32) of the included studies incorporated high GRV as an important indicator of EFI in critically ill children. The most common method to assess GRV is to use a syringe to draw back from the feeding device. However, although a high GRV is stated as the most important sign of gastro-intestinal intolerance (25), there is no common standard for the cut-off values. In 8 studies, a high GRV was commonly considered as ≥50% of the EN delivered in last 4 h (22, 26–31, 55). In other studies, the cut-off value ranges from 1/3 to 300% of the administered volume (34, 39, 42). Some scholars also developed specifical standards for continuous EN delivery, including 50% (32) or 200% (33) of the hour volume in continuous feeding, and 50% (38) of the previous total feeding amount. Another commonly used calculation of the cut-off value is based on body weight, which ranges from 3 ml/kg (34, 35) to 5 ml/kg (25, 33). Another measurement of GE is gastric ultrasound. By scanning gastric antrum diameters in a supine position and a right lateral decubitus position, the cross-sectional area of the antrum can be calculated. Then, different formula was chosen to calculate the gastric content volume according to the age of the child. In children ≤12 months, the formula proposed by Kim was suggested (44), and in children >11 months, the formula proposed by Spencer was used (43).

In addition, acetaminophen absorption test (AAT) can also be used to monitor GE. Since acetaminophen cannot be absorbed in stomach, the rise of blood acetaminophen concentration reflects gastric emptying and small intestinal absorption of acetaminophen (35, 56). A baseline acetaminophen level was obtained, and it was re-measured at 60mins from drug administration to calculate the area under the curve at 60 min (AUC60). AUC_60_ < 600 mcg min/ml is identified as delayed GE (35).

#### 3.3.2 GI symptoms

Although many GI symptoms were listed in the included studies as indicators of EFI, there were no detailed measurements for some of the symptoms, such as nausea, abdominal pain, increased bowl sounds, and gastrointestinal hemorrhage. Therefore, we only focused on five symptoms with specific measurements.

Diarrhea is another commonly used indictor of EFI, which was mentioned in 20 included studies. Majority (14/20) of the studies assessed diarrhea by measuring the frequency and nature of stools, and the other 6 studies also took the weight of stools and accompanying consequences into account. Loose and liquid stools were signs of diarrhea, and the threshold of frequency ranged from 3 to 6 times in a 24-h period. 5 studies considered 3 episodes as the threshold of diarrhea in critically ill children (35, 45–48). Only two studies (26, 57) considered the criteria for diarrhea according to the age of the child, and one of them suggested 8 liquid stools could be normal in infants younger than 3 months of age (26). When taking the weight of stools into consideration, 10 g/kg/day (39) and 20 cc/kg/day (51) was mentioned in two studies. Besides, negative fluid balance and inefficient use of antidiarrheal drugs were also considered as measurements of diarrhea in 4 studies (22, 29, 39, 55).

Vomiting was measured in 9 included studies. 88.89% (8/9) of the studies consider vomiting as 2–3 episodes with gastric content in 24 h (22, 31, 35, 40–42, 45, 55). Abdominal distension was measured in 7 included studies as an indicator of EFI. Abdominal grith and intra-abdominal pressure were assessed. Abdominal distension was considered when the abdominal grith increases 1.5–2 cm in 24 h or by more than 10% of the baseline girth (35, 42, 45, 46). It was also considered abdominal distension when intra-abdominal pressure was greater than 10 mmHg (40, 41). Another study argued that failure to improve vomiting symptom with GI motility drugs and the need to suspend feeding was an indicator of EFI (39). Constipation was also included in 5 studies, measuring by the days without stools. 2 of them set the threshold at 3 days (26, 52), while the others ranged from 24 h to 4 days (33, 53, 54). Besides, three articles described the measurements of aspiratory, two of them determined aspiratory according to the presence of stomach contents in the respiratory tract (40, 41). The other study assessed aspiration daily by the detection of gastric pepsin in tracheal secretions (48).

## 4 Discussion

### 4.1 Current EFI measurements are numerous and complex

In this article, we sought to synthesize and summarize major measurements of EFI in critically ill children. The articles included had identified multiple measurements of the indicators of EFI, and thus highlights the complexity of the measurements of EFI in pediatric critical illness care. We found that the EFI was commonly defined as anyone or a combination of the two aspects mentioned above: delayed GE and GI symptoms. However, different GI symptoms were considered as predictive indicators in different studies. We finally included 32 studies and there were 9 GI symptoms were covered in total. None of the studies included all of the GI symptoms and most of them only included 3–4 of them. Among the total 9 GI symptoms, only 5 of them was described in detail, which were summarize above. Besides, even the measurement of one same indicator varied from each other. In the 7 indicators summarized in this article, only one of them (AAT) was in agreement between two studies, and the other indicators were measured by different methods. High GRV was the most commonly used indicator of EFI, which was mentioned in 22 studies. Only 8 of the studies agreed on the same measurement of high GRV, and the remaining 14 studies listed a total of 12 additional measurements. Thus, we found it quite difficult to reach a uniform standard in this topic. Given the consensus on the benefits of adequate energy intake (14, 58), proper measurements of EFI to avoid unnecessary feeding interruptions is the focus of current research (37). The various measurements of EFI contributes to the difficulty in comparing the incidence of EFI, and also hinders the development of nutrition promotion research in critically ill children.

### 4.2 Current EFI measurements lack adaptability and necessity

Although current EFI measurements are numerous and various, they cannot provide targeted guidance in complex clinical situations. Although we had limited the age of critically ill children to 28 days—18 years, there were still differences in the gastrointestinal function development in children at different growth stages (59). Diarrhea was listed as an indicator over half of the included studies (20/34), while only 4 of them take the age or weight of children into consideration during the measurement (26, 33, 39, 49). The absolute limits to the number of normal bowel movements in children of different ages are difficult to define (60), so the World Health Organization also defines diarrhea as more frequently than normal situation for a person (61). Corresponding to that, 2 studies included the baseline level of bowel movements into the measurement of diarrhea (32, 49). Setting a low threshold for diarrhea may increase the risk of feeding interruptions and decrease the adequacy of nutrition intake. There is still a need to refine the criteria of diarrhea, taking full account of the age of children, bowel habits, and the influence of diarrhea.

Another controversial measurement is GRV. Similar to the results of this study, GRV is widely used as a surrogate marker of gastric emptying in a majority of PICUs (32, 36, 37). In a large multi-country studies conducted in Asia-Pacific and the Middle East (25), GRV was measured in 77% (36/47) of respondents from 18 different countries. However, despite being widely used, its clinical value has not been confirmed by the evidence. In a prospective cohort study in critically ill children, GRV failed to predict delayed gastric emptying, and related to slow EN advancement (35). Routine measurement of GRV in critically ill children is not recommended by the European Society of Pediatric and Neonatal Intensive Care (ESPNIC) as well (15). The measurement of GRV is frequently inaccurate due to the position of the feeding tube in the stomach, the feeding method, patient position, syringe sizes used, and the technique of aspiration (62, 63). Consequently, some researchers also begin to explore the feasibility of not routinely monitoring GRV. It is showed that routinely measure GRV was of questionable benefit (64), and not measuring GRV did not increase vomiting, ventilator-acquired pneumonia or necrotizing enterocoltis (65). When trying to change the long-standing and embedded practice of routinely measuring GRV, PICU nurse had significant fear around pulmonary aspiration (63). Therefore, future studies should explore individualized measures for different age stages and disease characteristics to accurately assess EFI, and advance EN intake to promote recovery in critically ill children.

### 4.3 More education and teamwork are needed

The management of EN delivery should be the responsibility of a nutrition support team (NST), including doctors, nurses, dietitians and pharmacists (66). The NST should use a stepwise algorithmic approach to advance EN in critically ill children, which must include the detection of EFI by bedside support (60). Due to the uniform measurements of each indicator, multiple indicators involved, and the loose definition of EFI, there are challenges in the measurements of EFI among this population (59). Thus, more focused nutrition education for NST should be highlighted (19). The understanding of EN measurements can be unified and further collaboration can be developed by written guidelines, multiprofessional nutrition rounds, and the continual auditing of practices (67). In the context of the continuous development of EFI measurements and the need to change clinical practice, the NST should work together to develop a viable protocol, which takes the opinions of parents into consideration (68). Currently, nurses play a vital role in the measure of EFI and the delivery of EN in PICU, and their knowledge can impact EN notably (63). A simple flow chart and education package were suggested to address concerns of junior nurses (65).

On the other hand, accurate measurement should be applied in critically ill children to assess delayed GE, which is the most common manifestation of gastric dysmotility in this cohort (56). Ultrasound is a non-invasive, non-interrupted measurement of EN. In a prospective observational study involving 64 critically ill children, gastric ultrasound improved that GRV appeared unreliable as a measure of gastric emptiness (43). But the performance of ultrasonography is positionally dependent and is not appropriate in children with special positioning, such as the prone position. AAT was also reported in previous studies, while it can only be administered to eligible children (35). The need for additional blood samples may also increase the pain and economic burden of children. Electrical impedance method is another measurement of GE, which showed encourage effect in the monitoring of EFI in non-invasive critically ill patients (69). Developing accurate bedside measures for gastric emptying are highly desirable and need to be further investigated.

## 5 Strengths and limitations

The literature was systematically searched and screened following an *a priori* protocol, and guidelines published by the Joanna Briggs institute. In highlight these findings, we also acknowledged some limitations of this review and across the studies examined. The definition of EFI was various in included articles, which was not summarized in detail as it was beyond the scope of this review. It may add the difficulty of the understanding of EFI. Another limitation was that only 5 GI symptoms were summarized in this article, due to the lack of specific information of other GI symptoms. Future researchers can develop further studies of EFI measurements by providing more detailed information.

## 6 Conclusions

The current paper reviewed the numerous measurements of EFI in critically ill children, which was categorized by 7 indicators of EFI. The summarized data provided insight into how EFI was assessed, and highlighted the complexity of the measurements. Importantly, the various measurements applied in different studies increased the difficulty of integrating the results. It highlighted the necessary to develop individual measurements of EFI. We suggested that the age of children and disease condition to be taken into consideration. Further studies can also investigate accurate and objective physiological measurements of EFI to advance EN and improve outcomes in critically ill children.
