# Supplementary material for: Measurements of enteral feeding intolerance in critically ill children: a scoping review
Source: Front Pediatr. 2024 Oct 10;12:1441171. doi: 10.3389/fped.2024.1441171 (PMC11499133; doi:10.3389/fped.2024.1441171)
Supplement: Supplementary file 1 [file Table1.docx]

Table 1 Indicators and corresponding measures of EFI in critically ill children

| Author | Region (year) | Journal | Objectives | Design | Population | Indicators of EFI and Measures |
| --- | --- | --- | --- | --- | --- | --- |
| Eveleens et al.^22^ | Netherlands (2020) | Clinical Nutrition | To investigate the definitions, prevalence, predictors and outcomes of FI in critically ill children | Systematic Review | Critically ill children | 1. High GRV:≥50% of the EN delivered in the last 4 h 2. Diarrhea: ≥4 times loose or liquid stool, with negative fluid balance in 24 h period 3. Vomiting: ≥2 times with gastric content in 24 h period |
| Ying et al.^25^ | China (2023) | European Journal of Pediatrics | To understand the characteristics of children with FI and identify the factors predicting FI in critically ill children | Retrospective Cohort | Critically ill children | 1. High GRV: ≥50% of the EN delivered in the last 4 h 2. Diarrhea: ≥4 times loose or liquid stool, with negative fluid balance in 24 h period 3. Vomiting: ≥2 times with gastric content in 24 h period |
| Pérez  et al. ^27^ | Spain (2022) | Journal of Pediatric Gastroenterology and Nutrition | To assess the safety of enteral nutrition in children on extracorporeal membrane oxygenation. | Retrospective Cohort | Pediatric patients on extracorporeal membrane oxygenation | 1. High GRV: ≥ 50% of the EN delivered in the last 4 h 2. Diarrhea: ＞8 liquid stools in infants <3 months of age,＞4 liquid stools in 3-12-month-old children,＞2 liquid stools in children >12 months 3. Abdominal Distension: increased abdominal circumference on the sagittal plane 4. Constipation: ≥3 days without bowel movement after the start of EN |
| López-Herce et al. ^28^ | Spain (2008) | European journal of clinical nutrition | To study the risk factors for gastrointestinal complications related to enteral nutrition in critically ill children. | Prospective Cohort | Critically ill children | High GRV:≥ 50% of the EN delivered in the last 4 h |
| López-Herce et al. ^29^ | Spain (2008) | Nutrition journal | To analyze the characteristics of enteral nutrition and its tolerance in the critically ill child with shock and to compare this with non-shocked patients. | Prospective Cohort | Critically ill children | High GRV:≥ 50% of the EN delivered in the last 4 h |
| van Waardenburg et al.^30^ | Netherlands (2009) | Clinical Nutrition | Explore the effect of protein and energy-enriched infant formulas in achieving nutritional targets. | RCT | Critically ill children | 1. High GRV: ≥50% of the EN delivered in the last 4 h 2. Diarrhea: ≥ 4 times loose or liquid stool, with negative fluid balance in 24 h period |
| Sánchez et al. ^31^ | Spain (2007) | Nutrition | To compared the tolerance of early and late transpyloric enteral nutrition in critically ill children. | Prospective Cohort | Critically ill children | 1. High GRV: ≥50% of the EN delivered in the last 4 h 2. Diarrhea: ≥5 loose stools per day |
| Yuqing et al. ^32^ | China (2021) | Journal of Nursing Science | To compare the sensitivity and specificity of different process assessment indicators or combinations of indicators in the diagnosis of feeding intolerance in critically ill children | Retrospective Cohort | Critically ill children | 1. High GRV: ≥ 50% of the EN delivered in the last 4 h 2. Vomiting: ≥ 2 times with gastric content in 24 h period 3. Abdominal Distension: increase in abdominal circumference ≥10% on 2 consecutive occasions within 24 hours |
| Chiusolo et al. ^36^ | Italy (2020) | Pediatric gastroenterology, hepatology & nutrition | To assess the effectiveness and safety of amoxicillin/clavulanate (A/C) to treat EN intolerance. | Quasi-experimental Study | Critically ill children | 1. High GRV: in continuous EN delivery, GRV≥50% of the volume/h at least 3 consecutive evaluations, in intermittent EN delivery, GRV≥50% of the bolus volume at least 3 consecutive evaluations 2. Diarrhea: ≥3 liquid stools/day in patients with previous normal stool and/or increase of ≥50% of the number of liquid stools |
| Bartkowska-Śniatkowska et al. ^37^ | Poland (2015) | Anaesthesiology Intensive Therapy | In the present study, methods for nutritional treatments in critically ill children are presented, depending on the clinical situation. | Consensus | Critically ill children | 1. High GRV: in intermittent EN delivery, GRV should be measured before each bolus or every 4h, GRV > 5ml/kg or over 50% of volume of the portion administered or 200ml (in children with body weight ＞40kg); in continuous EN delivery, GRV ≥200% of hour volume 2. Diarrhea: ≥4 loose stools/day 3. Constipation: ＞48 hours without feces after the start of EN |
| Liauchonak et al. ^34^ | USA (2023) | Nutrition in Clinical Practice | To examine whether revising the EN intolerance definition of an algorithm would decrease EN interruptions and improve nutrient delivery in critically ill children. | Quasi-experimental Study | Critically ill children | High GRV: for patients >50 kg, GRV ＞250ml,  for patients <50 kg, GRV ＞3 ml/kg |
| Martinez et al. ^39^ | USA(2017) | Journal of Parenteral and Enteral Nutrition | To examine the correlation between (a) bedside EN intolerance assessments, including gastric residual volume (GRV); (b) delayed GE; and (c) delayed EN advancement | Prospective Cohort | Critically ill children | 1. High GRV: GRV >3 mL/kg or >150 Ml 2. Acetaminophen Absorption Test: A baseline acetaminophen level was obtained, and it was re-measured at 60±5mins from acetaminophen administration 3. Diarrhea: ≥3 episodes of loose or liquid stool in a 24-hour period 4. Vomiting: ≥ 2 times with gastric content in 24 h period 5. Abdominal Distension: 2 or more increases in abdominal girth in a 24-hour period |
| Veldscholte et al. ^60^ | Canada (2023) | Journal of Pediatric Gastroenterology and Nutrition | To investigated the course of several gastrointestinal biomarkers and their association with EN advancement longitudinally during pediatric intensive care unit admission. | RCT | Critically ill children | High GRV: ≥50% of delivered EN over 24 hours |
| Solana et al. ^55^ | Spain (2023) | Nutrients | To describe the characteristics of Enteral nutrition interruption in the pediatric intensive care unit . | Observation | Critically ill children | High GRV: ≥50% of delivered EN over 24 hours |
| Xianrong et al. ^38^ | China (2020) | The heart surgery forum | To explore the effects of breast milk feeding and formula milk feeding on infants after cardiac surgery in the cardiac intensive care unit. | Retrospective Cohort | Infants after cardiac surgery in ICU | 1. High GRV: in continuous EN delivery, GRV more than 50% of the previous total feeding amount 2. Diarrhea: defecation multiple times a day, mostly in the morning or after feeding, and mushy and watery stool, with a pungent odor |
| Wong et al. ^25^ | Singapore (2016) | Asia Pacific journal of clinical nutrition | To survey the nutrition practices and perspectives of paediatric intensivists and dieticians in Asia-Pacific and the Middle East. | Observation | Critically ill children | High GRV: GRV >5 mL/kg or in > 50% of the last feed volume |
| Yanqin et al.^35^ | China (2018) | Nutrition | To evaluate nutrition effects and tolerance of a PE-formula compared with the standard formula (S-formula) in infants in the first 5 days after congenital heart surgery. | RCT | Infants following congenital heart surgery |  |
| Shuangyu et al. ^49^ | China (2023) | China journal of Primary Medicine and Pharmacy | To explore the intervention effect of enteral nutrition tolerance management program in children with severe sepsis | RCT | Critically ill children | 1. High GRV: in continuous EN delivery, GRV> 40% of the previous amount of milk pumped 2. Diarrhea: ≥1 time every 12 hours 3. Vomiting: ≥1 time every 12 hours 4. Abdominal Distension: intra-abdominal pressure over 10 mmHg (1 mmHg=0.133 kPa) 5. Aspiration: Suction of stomach contents from the respiratory tract. |
| Xianmin et al. ^50^ | China (2018) | China Medical Herald | To investigate the effect of enteral nutrition tolerance management program on early EN tolerance in children with severe sepsis | RCT | Critically ill children | 1. High GRV: in continuous EN delivery, GRV> 40% of the previous amount of milk pumped 2. Diarrhea: ≥1 time every 12 hours 3. Vomiting: ≥1 time every 12 hours 4. Abdominal Distension: intra-abdominal pressure over 10 mmHg (1 mmHg=0.133 kPa) 5. Aspiration: Suction of stomach contents from the respiratory tract. |
| Huimin et al.^33^ | China (2022) | China Medical University | By compiling and applying the questionnaire of knowledge, attitude and practice (KAP) of nurses in Pediatric Intensive Care unit (PICU) on enteral nutrition feeding intolerance, to explore the current level and influencing factors. | Quasi-experimental Study | Critically ill children | 1. High GRV: GRV >1/3 of the previous feeding volume 2. Diarrhea: ≥6 loose stools per 24 hours 3. Vomiting: ≥3 times/day 4. Abdominal Distension: abdominal circumference increase > 1.5 cm in 24 hours, with intestinal type |
| Valla et al. ^41^ | France (2022) | Frontiers in Pediatrics | To explore the effect of point-of-care ultrasound among pediatric intensivists. | Prospective Cohort | Critically ill children | High GRV: Scan gastric antrum larger and shorter diameters in a supine position and a right lateral decubitus position, to calculate the cross-sectional area of the antrum and extrapolating the gastric content volume based on the formula proposed by Spencer. The gastric content was described as ‘empty’ or ‘full with liquid’ or ‘full with both solid and liquid’. |
| Kaile et al.^40^ | China (2023) | Chinese Pediatric Emergency Medicine | To review the progress on feeding intolerance and the relationship between gastric residual volume and feeding intolerance. | Review | Critically ill children | 1. High GRV: In children ≤12 months, the formula proposed by Kim is more appropriate. The gastric volume can be delivered into 3 levels according to the presence of fluid in the gastric sinus in supine position or the right lateral decubitus position 2. Acetaminophen Absorption Test: A baseline acetaminophen level was obtained, and it was re-measured at 60±5mins from acetaminophen administration to calculate the area under the curve at 60 minutes (AUC_60_). AUC_60_<600mcg·min/ml is identified as delayed GE. |
| Hamilton et al. ^43^ | USA (2014) | Pediatric Critical Care Medicine | To evaluate the impact of implementing an enteral nutrition algorithm on achieving optimal enteral nutrition delivery in the PICU | Quasi-experimental Study | Critically ill children | 1. Diarrhea: ≥3 episodes of loose or liquid stool in a 24-hour period 2. Vomiting: ≥ 2 times with gastric content in 24 h period 3. Abdominal Distension: 2 or more increases in abdominal girth in a 24-hour period |
| Kumar et al. ^44^ | India (2023) | Indian journal of pediatrics | To compare the time taken to reach the target calories and proteins by protocol based "continuous tube feeding" and "intermittent tube feeding " in critically ill children. | RCT | Critically ill children | 1. Diarrhea: ≥3 episodes of loose or liquid stool in a 24-hour period 2. Abdominal Distension: >10% increase from baseline girth |
| Weckwerth et al. ^45^ | USA(2004) | Nutrition in Clinical Practice | To describes commonly used monitors for tolerance to enteral nutrition for infants and children and discusses pertinent data relevant to practice. | Review | Critically ill children | Diarrhea：≥3 episodes of loose or liquid stool in a 24-hour period |
| Meert et al. ^46^ | USA (2004) | Chest | To determine the effect of feeding tube position (gastric vs small bowel) on adequacy of nutrient delivery and feeding complications, including microaspiration, in critically ill children. | RCT | Critically ill children | 1. Diarrhea：≥3 episodes of loose or liquid stool in a 24-hour period 2. Aspiration：Aspiration was assessed by the detection of gastric pepsin in tracheal secretions. Tracheal secretions (0.1 to 0.5 mL) were collected from the endotracheal tube daily without the use of saline solution lavage |
| Panchal et al. ^57^ | USA (2016) | Journal of parenteral and enteral nutrition | To evaluate the safety of enteral feeding in children receiving vasoactive agents . | Retrospective Cohort | Critically ill children | Diarrhea：≥3 loose watery stool in a day; for infants, it is defined as the passage of stool at least 1.5-2 times more frequent than the baseline level |
| Brown et al.^69^ | USA (2012) | ICAN Infant, Child, & Adolescent Nutrition | To explore that a protocolized continuous gastric EN approach would decrease time to goal feeding rate and calories. | Quasi-experimental Study | Critically ill children | Diarrhea：≥6 loose stools per 24 hours |
| Jacobs et al. ^48^ | USA (2013) | Pediatric Critical Care Medicine | To evaluate the impact of such an approach on the alteration of plasma phospholipid fatty acid concentrations. | RCT | Critically ill children | Diarrhea：> 20 cc/kg/ day of stool |
| Solana et al. ^51^ | Spain (2021) | Nutrition | To analyze the nutritional status, NS characteristics, macronutrient supply, and associations between NS and outcomes in critically ill children in Spain. | Prospective Cohort | Critically ill children | Constipation：≥3 days without bowel movement after the start of EN |
| Marino et al. ^52^ | UK (2019) | Journal of human nutrition and dietetics | To characterise the use of a PEF amongst critically ill infants in two paediatric intensive care units . | Retrospective Cohort | Critically ill children | Constipation：≥4 days without stools |
| Brown et al. ^53^ | USA (2019) | Journal of parenteral and enteral nutrition | To compare the effectiveness and safety of C-GF vs B-GF in intubated pediatric patients | Quasi-experimental Study | Critically ill children | Constipation：＞24 hours without stools |
